# Does Applicability Domain Exist in Microarray-Based Genomic Research?

**DOI:** 10.1371/journal.pone.0011055

**Published:** 2010-06-10

**Authors:** Li Shao, Leihong Wu, Hong Fang, Weida Tong, Xiaohui Fan

**Affiliations:** 1 Pharmaceutical Informatics Institute, College of Pharmaceutical Sciences, Zhejiang University, Hangzhou, China; 2 Z-Tech Corporation, an ICF International Company at the National Center for Toxicological Research/United States Food and Drug Administration, Jefferson, Arkansas, United States of America; 3 National Center for Toxicological Research, United States Food and Drug Administration, Jefferson, Arkansas, United States of America; University of East Piedmont, Italy

## Abstract

Constructing an accurate predictive model for clinical decision-making on the basis of a relatively small number of tumor samples with high-dimensional microarray data remains a very challenging problem. The validity of such models has been seriously questioned due to their failure in clinical validation using independent samples. Besides the statistical issues such as selection bias, some studies further implied the probable reason was improper sample selection that did not resemble the genomic space defined by the training population. Assuming that predictions would be more reliable for interpolation than extrapolation, we set to investigate the impact of applicability domain (AD) on model performance in microarray-based genomic research by evaluating and comparing model performance for samples with different extrapolation degrees. We found that the issue of applicability domain may not exist in microarray-based genomic research for clinical applications. Therefore, it is not practicable to improve model validity based on applicability domain.

## Introduction

Emerging technologies such as gene expression microarrays offer unprecedented opportunities for clinical cancer research [1], [2], [3]. A decade of intensive research into developing predictive models that are capable of dividing patients into clinically relevant groups has yielded a number of demonstrable successes. Two primary examples of this are models to divide patients into groups with differing event-free survival [4], [5], [6] and to identify groups of patients with different expected response to therapy [7], [8], [9].

However, challenges remain in this field [10], [11], [12]. The validity of some models has been questioned due to their failure to clinical validate using independent samples. A recent example is a model for breast cancer prognosis built with two genes by that Reid *et al*. [11] that could not be validated by other investigators [13]. From a statistical point of view, as reviewed by Simon [14], this type of prediction is a complicated problem and many factors, such as gene selection rules, sample resubstitution approaches, sample size concerns, and classification methods are involved. Fortunately, some of these factors have been extensively investigated and are incorporated as “best practices” in the research community. Ambroise, *et al.* demonstrated that the test/validation set must play no role in the gene selection process for unbiased prediction results to be obtained [15]. Ransohoff, *et al.* [16] emphasized that over-fitting should be explicitly ruled out by reproducibility assessment early on, otherwise further research (that is, additional steps in the validation process) would be unwarranted and wasteful.

The importance of applicability domain (AD) [17] (i.e., the scope and limitations of a model) has long been discussed and emphasized in other research fields such as quantitative structure activity (property) relationship (QSAR) analysis [17], [18], [19], [20]. AD in QSAR emphasizes that no matter how robust, significant and validated a model may be, it cannot be expected to reliably predict the modeled property for the entire universe of chemicals. Therefore, before a model is put into use for screening chemicals, its domain of application must be defined and predictions for only those chemicals that fall in this domain may be considered reliable [17].

However, the AD effect in genomic research has not been fully understood. The carcinoembryonic antigen (CEA) experience [21], [22] from 40 years ago, where non-reproducible results were obtained largely due to the variation among the test sets in terms of the ‘spectrum’ of disease, initially implied the vital importance of selecting appropriate validation samples in order to reliably assess the reproducibility of statistical modeling results. Nevertheless, this issue has not yet been adequately addressed by the microarray-based ‘class prediction’ research community until now.

Two sources of divergence between training and validation samples exist: clinical differences such as diversity in cancer subtype, drug response, or prognosis, and genomic differences, or differences between gene expression patterns observed in the training and validation samples. We have undertaken a comprehensive investigation of the role of genomic differences in predictive model validation to determine if a genomic AD exists for microarray based ‘class-prediction’ modeling. We hypothesize that validation samples that more closely resemble the genomic space defined by the training set might be more likely to have accurate predictions than validation samples that significantly diverge from the genomic space defined by the training set.

A statistical measure called domain extrapolation [23] has been introduced to assess the genomic AD issue. Domain extrapolation is a measurement embedded in the model to place the patients in different groups according to their extrapolation degree. The role of genomic AD in microarray-based ‘class-prediction’ will be tested using three large-scale cancer datasets with six clinical endpoints [24] contributed to the MAQC Consortium and three prognostic datasets [4], [25], [26]. To mimic the real world clinical situation, each dataset was divided into two sets, i.e., a training and validation set. We developed the domain extrapolation in the training set and followed with the assessment of its correlation with the model's predictive ability in the validation set. To the best of our knowledge this is the first attempt to systematically evaluate the issue of genomic AD in microarray-based genomic research.

## Materials and Methods

### Datasets

Nine datasets, including three large-scale cancer datasets - breast cancer (BR) [27], multiple myeloma (MM) [28] and neuroblastoma (NB) [29] with six clinical endpoints contributed to the MAQC Consortium [24] and three datasets used in previously published prognostic modeling research [4], [25], [26], were selected and utilized in this study. A concise summary of the datasets is given in Table 1. More information about these datasets can be found from the main paper of MAQC phase II [24] and the original papers [4], [25], [26].

**Table 1:** A concise summary of the datasets.

Data Set code	Number of channels (type)	Endpoint Description	Endpoint Code	Sample Size		Number of events (%)	
				Training	Validation	Training	Validation
BR	1 (Affymetrix U133A)	Treatment Response	BR-pCR	130	100	0.34 (33/97)	0.18 (15/85)
			BR-erpos	130	100	1.60 (80/50)	1.56 (61/39)
MM	1 (Affymetrix U133Plus2.0)	Overall Survival Milestone Outcome	MM-OS	340	214	0.18 (51/289)	0.14 (27/187)
		Event-free Survival Milestone Outcome	MM-EFS	340	214	0.33 (84/256)	0.19 (34/180)
NB	2 (Agilent NB Customized Array)	Overall Survival Milestone Outcome	NB-OS	246	177	0.32 (59/187)	0.28 (39/138)
		Event-free Survival Milestone Outcome	NB-EFS	246	193	0.65 (97/149)	0.75 (83/110)
NHL	2 (Lymphochip)	Overall Survival Milestone Outcome	NHL	160	80	1.22 (88/72)	1.67 (50/30)
BRC	2 (Agilent Hu25K microarrays)	5-year metastasis-free survival	BRC	78	19	0.77 (34/44)	1.71 (12/7)
HCC	1 (Affymetrix)	1-year recurrence-free survival	HCC	33	27	0.57 (12/21)	0.42 (8/19)
Control	2 (Agilent NB Customized Array)	Positive control	NB-PC	246	231	1.44 (145/101)	1.36 (133/98)
	1 (Affymetrix U133Plus2.0)	Positive control	MM-PC	340	214	1.33 (194/146)	1.89 (140/74)
	2 (Agilent NB Customized Array)	Negative control	NB-NC	246	253	1.44 (145/101)	1.30 (143/110)
	1 (Affymetrix U133Plus2.0)	Negative control	MM-NC	340	214	1.43 (200/140)	1.33 (122/92)

Briefly, each of the three large-scale cancer datasets has two endpoints, including the treatment response (BR-pCR and BR-erpos), the event-free survival (NB-EFS and MM-EFS) and the overall survival (NB-OS, MM-OS) which are related to cancer prognosis. The other three datasets are related to the survival of non-hodgkin lymphoma (NHL), breast cancer (BRC) and hepatocellular carcinoma (HCC). To simulate the real-world clinical application of genomic studies, two independent populations of patients for each dataset created by the MAQC Consortium or by the original researchers are retained in this study as the training and validation sets. The sample size for the training set varies between 33 and 340 and the ratio of positive events to negative events is from 0.18 to 1.60 while the validation sets range in size from 19 to 214.

Moreover, two positive (NB-PC, MM-PC) and negative (NB-NC, MM-NC) control endpoints available from the MAQC project were also included in this study, which are necessary to assess the performance of the clinically relevant endpoints against the theoretical maximum and minimum performance provided by the controls. The NB-PC and MM-PC were derived from the NB and MM datasets, respectively, with the endpoints denoted by the gender while the endpoints for the NB-NC and MM-NC were generated randomly.

### Applicability domain (AD)

AD [30] of a microarray-based predictive model can be stated as the genomic or biological space, knowledge or information defined by the training set with which the predictive model has been developed, and thus for which it is applicable to new patients. Ideally, the model should only be used to make predictions within that domain by interpolation not outside that domain by extrapolation. In this study, we focus exclusively on genomic AD, or quantifying the degree of extrapolation or difference between the genomic space defined by the training set and each validation sample. The genomic AD of a model was defined based on the Euclidean distance [30] using the method shown as follows.

Suppose there is a training set (*X*) that contains *n_1_* samples and *p* genes. We can define the mean value (*m_j_*) and standard deviation (*s_j_*) for each gene *j* (*j* = 1, 2,…,*p*) across the entire dataset as 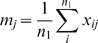 and 
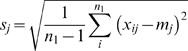
, where *x_ij_* is the expression value of gene *j* for individual *x_i_* (*i* = 1,2,…,*n_1_*). For any test set (*Y*) with *n_2_* samples and *p* genes, let *y_ij_* denote the expression value of the *j*th gene in *i*th (*i* = 1, 2,…, *n_2_*) sample. Then, the distance (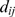) beyond the training domain for the unknown sample *y_ij_* for component *j* can be calculated by
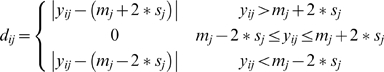
(1)


Thus, the total percentage of extrapolation *d_i_* for *i*th (*i* = 1, 2, …, *n_2_*) sample of the test set could be obtained as follows:
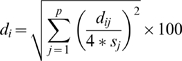
(2)


For each individual *y_i_*, *d_i_* is greater than or equal to 0, with 0 indicating samples lying in domain. The larger *d_i_* the more distantly away a sample removed an individual is from the training domain. For the sake of simplicity, the extrapolation degree *d_i_* has been grouped into four categories: in domain (*d_i_ = 0)*, less than 10% out of domain (*d_i_* 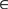 0–10), 10–20% out of domain (*d_i_* 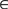 10–20), and more than 20% out of domain (*d_i_*>20).

### Statistical analysis

**Figure 1:**
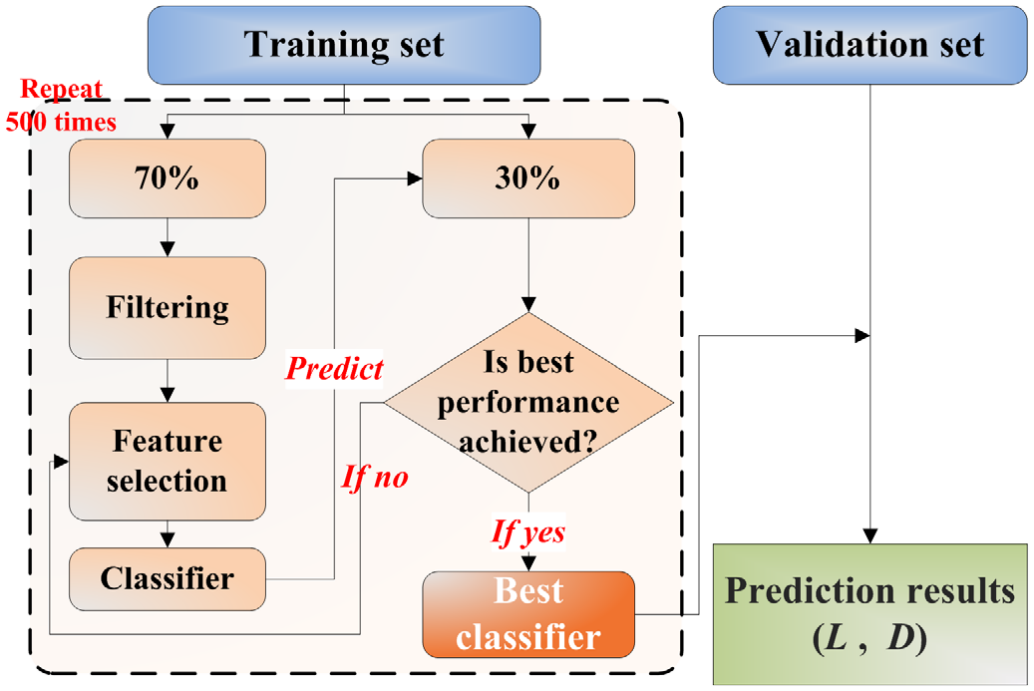
Detailed workflow for the statistical analysis.

As illustrated in the workflow shown in **Figure 1**, the analysis protocol starts on the left side of the graph by developing the best classifier based on the training set and ends on the right side by making a prediction about each individual in the validation set, where the predicted labels and corresponding extrapolation degrees are recorded in matrices *L* and *D*, respectively. To ensure statistical validity, the procedure was repeated 500 times, resulting in 500 different classifiers from the training sets and 500 predictions for each individual in the validation sets. Detailed information about model construction procedures is provided in Figure S1. In this study, nearest-centroid (*NC*) [4], k-nearest neighbor (*kNN*) [31] and support vector machines (*SVM*) [32] were used as classification algorithms.

Based on the 500-run results, we further divided the predictions in matrix *L* into subsets according to the category of extrapolation degrees (i.e., “in domain”, “<10% out of domain”, “10–20% out of domain”, and “>20% out of domain”) deposited in *D*. The prediction performance (as measured by Matthews correlation coefficient (MCC)[33]) for samples in each subset provides an illustration of model performance versus the stepwise increase of extrapolation degree. The Matthews Correlation Coefficient (MCC) is defined as:
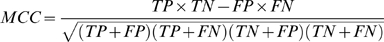
(3)


Where 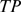 is the number of true positives, 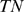 is the number of true negatives, 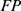 is the number of false positives and 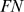 is the number of false negatives. MCC varies between −1 and +1 with 0 corresponding to random prediction.

## Results

**Figure 2:**
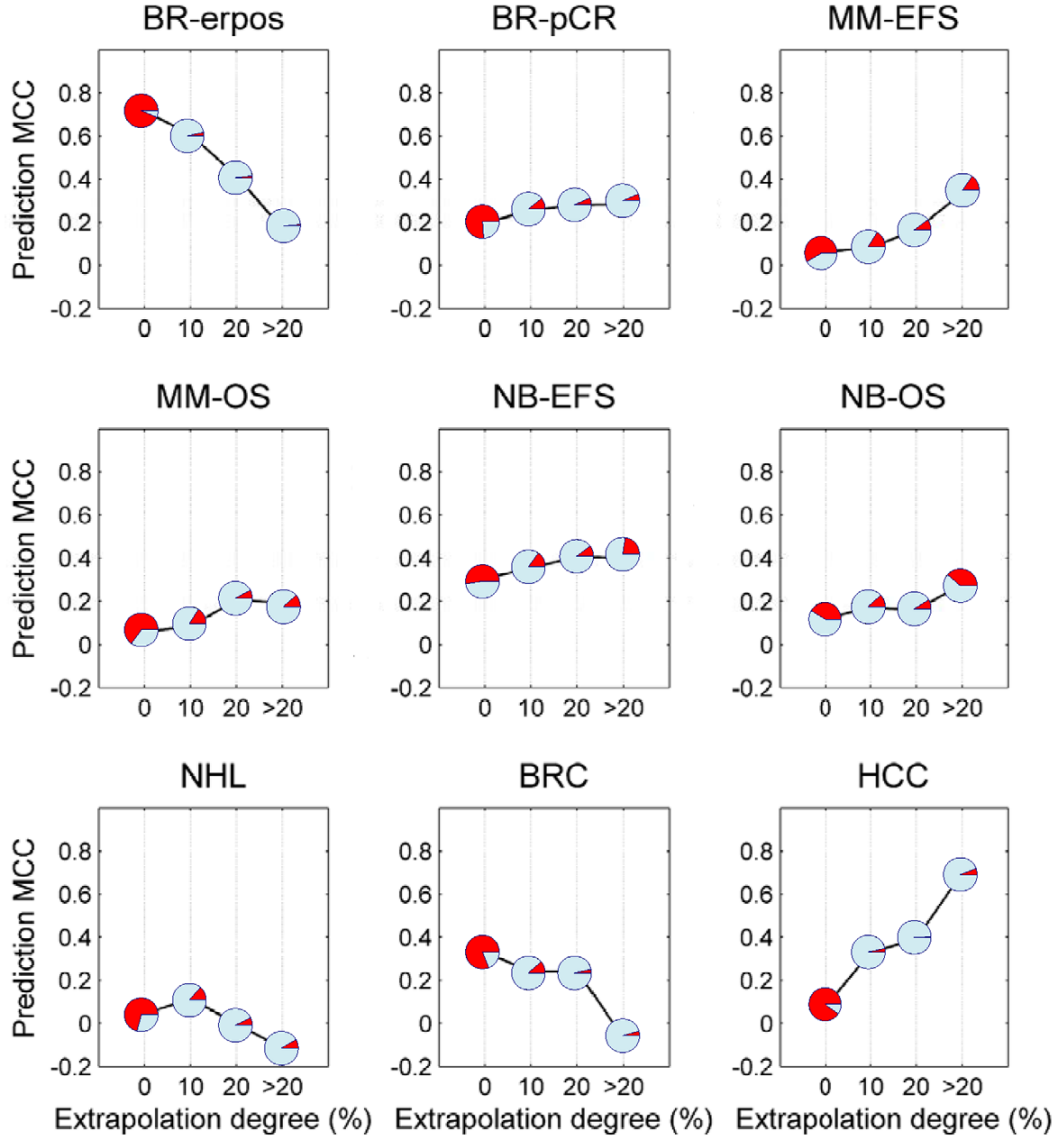
Prediction MCC as a function of extrapolation degree for nine datasets using *kNN* classifier. The proportion of red in each pie chart represents the proportion of total validation set samples contained in that extrapolation degree category. Here ‘0’, ‘10’, ‘20’ and ‘>20’ in the X-axis mean ‘In domain’, ‘0–10% out of domain’, ‘10–20% out of domain’ and ‘more than 20% out of domain’, respectively.

**Figure 3:**
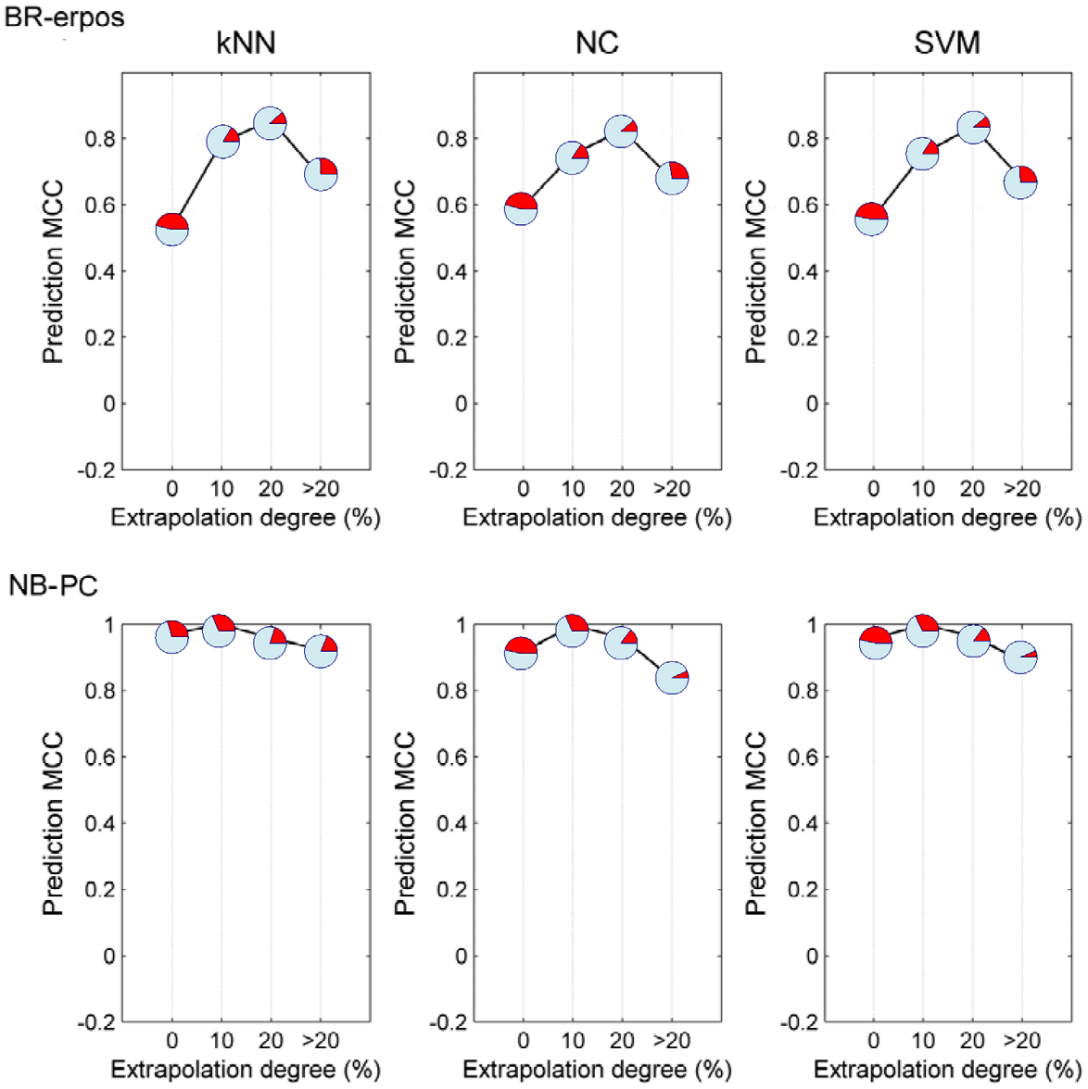
Adjusted prediction MCC versus extrapolation degree for BR-erpos and NB-PC. Three classification algorithms including *NC*, *kNN* and *SVM* are used, and the percentage of samples in each interval out of domain is adjusted to more than 10%. The proportion of red in each pie chart represents the proportion of total validation set samples contained in that extrapolation degree category. Here ‘0’, ‘10’, ‘20’ and ‘>20’ in the X-axis mean ‘In domain’, ‘0–10% out of domain’, ‘10–20% out of domain’ and ‘more than 20% out of domain’, respectively.

The prediction MCC as a function of extrapolation degree category for the nine datasets using *kNN* is shown in **Figure 2** **,** using *NC* in **Figure S2,** and using SVM in **Figure S3**. In each of the graphs, the red section of the pie-charts representing the data points show the proportion of the total testing set contained in that category of extrapolation degree. Generally, no significant impact on AD is observed, as evidenced by the slight increase in MCC for samples lying out of domain compared to those in domain for most datasets except BR-erpos. In BR-erpos validation set, fewer than 2% of the samples were in each of the 10–20% extrapolation and >20% extrapolation. We re-analyzed the results by distributing samples into the training and validation sets so that each of these categories has around 10% of the samples in the validation set. This modification resulted in the disappearance of any significant effect of extrapolation degree on MCC for each of the classification algorithms (**Figure 3**).

**Figure 4:**
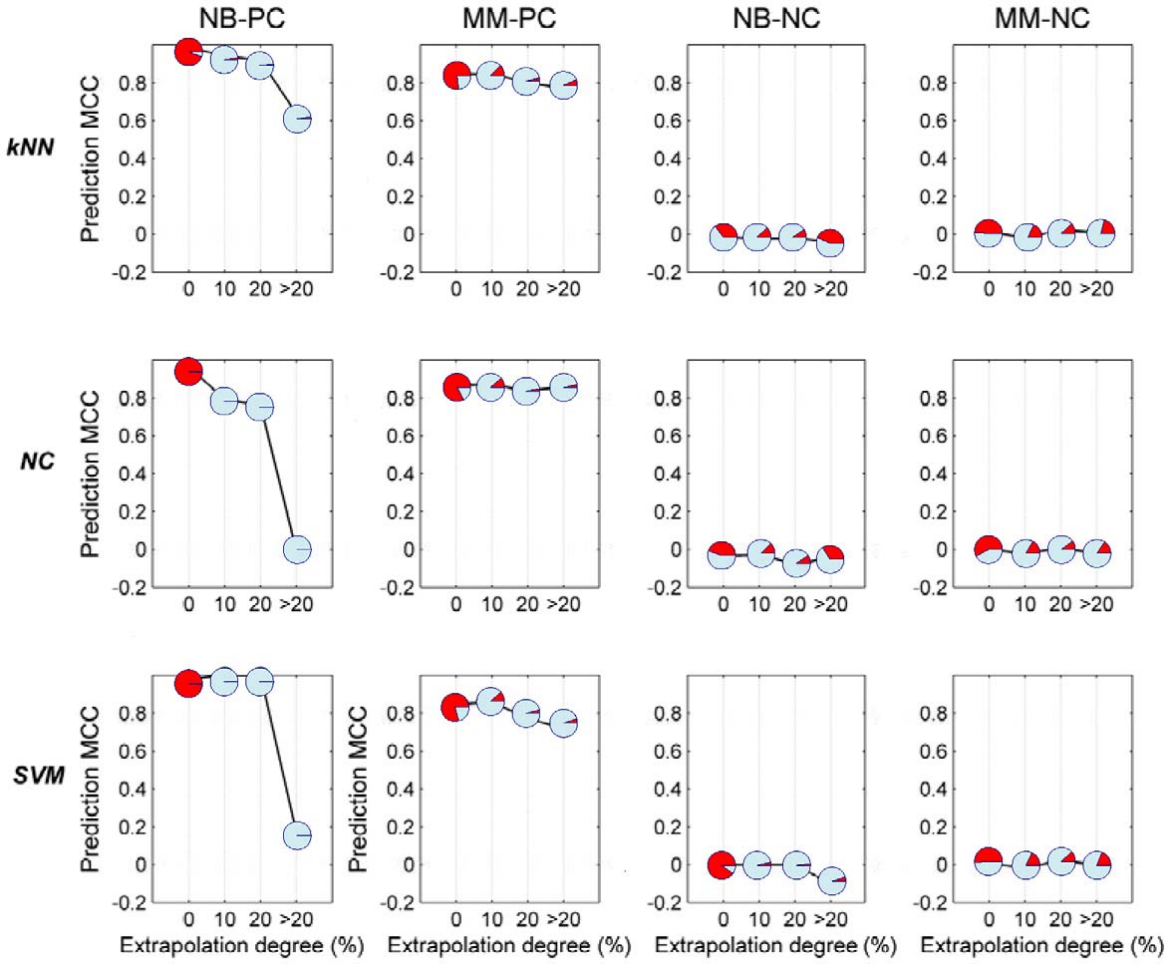
Prediction MCC versus extrapolation degree for positive and negative control datasets. Three classification algorithms including *NC*, *kNN* and *SVM* are used. The proportion of red in each pie chart represents the proportion of total validation set samples contained in that extrapolation degree category. Here ‘0’, ‘10’, ‘20’ and ‘>20’ in the X-axis mean ‘In domain’, ‘0–10% out of domain’, ‘10–20% out of domain’ and ‘more than 20% out of domain’, respectively.

In order to accurately assess the upper and lower bounds of performance and provide a point of reference for the prognostic datasets, two positive control datasets (i.e., NB-PC and MM-PC) and two negative control datasets (i.e., NB-NC and MM-NC) were also investigated. **Figure 4** demonstrates the results for these datasets for each of the three different classification methods used. The decrease in model performance is nearly negligible for MM-PC, while model performance drastically deteriorated for NB-PC when samples lay more than 20% degree out of domain. Considering that more than 95% of the samples lie in the domain for NB-PC, the same strategy utilized above was also used to ensure a larger percentage of samples in each interval, which yielded significantly smoothed curves shown in **Figure 3**. Additionally, negligible variation of model performance is observed for negative control datasets, where NB-NC and MM-NC (**Figure 4**) supports these conclusions.

## Discussion

Although differences in genome-wide gene expression patterns has been suggested previously as a possible reason for some failed applications of microarray based ‘class-prediction’ models to validate clinical models [21], [22], this is the first comprehensive investigation to identify whether genomic AD is truly a concern for microarray-based predictive modeling. Our results strongly suggest that genomic AD may not exist for clinical microarray-based genomic research. In other words, the expectation of improving model validity based on genomic AD is not practical in microarray-based genomic research.

The exact reasons for the negligible impact of genomic AD on model performance is beyond the scope of this study. However, two aspects may provide some explanation to this phenomenon: first, the genomic AD created by the training set may contain much more variability than is represented by the signature genes selected in the predictive models; second, the domain definition method utilized in this study might not be sensitive enough to capture the difference between samples inside and outside the domain. In clinical applications, model AD should be defined in not only a statistical or genomic but also a biological way, representing the training domain defined by parameters selected in statistical models and *a priori* clinical information. In other words, the insignificant impact of a genomic AD for complex endpoints does not negate the importance of considering clinical parameters when predicting independent validation samples. A simple but important example is that the information of cancer subtype must be considered before model development and use to ensure the reliability of any prediction, since the prognosis may differ significantly between subtypes [34].

As an interesting side note to this study, the three well known classification methods, i.e. *kNN*, *NC* and *SVM*, used in this study (with corresponding results provided in **Figure 2** and **Figures S2 and S3**, respectively) gave very similar prediction performance for samples with different extrapolation degrees. This offers further evidence for the lack of significant differences among a large number of classification methods reported for microarray applications in terms of the predictive performance[35], a conclusion also proposed by the newly-finished community-wide study, MAQC-II [24].

In conclusion, our study found that the applicability domain may not exist for microarray based clinical genomic research, and that predictive model performance did not depend on a measurement of distance between a validation sample and the training set used to create the model. Because of this, a strategy of considering applicability domain to increase model validation performance is unlikely to be successful. However, the negative conclusion in this study does not deny the importance of considering *a priori* clinical information associated with prognosis such as cancer subtype and estrogen receptor status for breast cancer patients before making an individual prediction, the importance of which has already been proposed by other studies.

## Supporting Information

Figure S1 Detailed model construction procedures. The construction of the best classifier is shown as follows (see the superscripts in this figure): 1. Stratified random sample splitting - We use the 70/30 splitting, where the 70% samples are for classifier construction, and the resulting classifier is then used to predict the 30% samples to obtain the prediction performance of the classifier. To ensure statistical validity, we repeat this procedure 500 times, resulting in 500 different classifiers. 2. Filtering - This step is to generate an initial pool of probesets for further analysis. Specifically, the original pool of probesets is firstly sorted by the absolute signal-to-noise (SN) ratio, and then the 200 top ranked probesets are retained for further analysis. 3. Feature selection - We apply a sequential selection method, with the best performed probeset being sequentially added into the model to develop a classifier, which is then evaluated on the 30% samples. The process is repeated by incrementally adding one probeset at a time to generate more classifiers. 4. Classifier selection - For classifier *i* (*i* corresponds to the number of probesets selected in the classifier), if the performance MCC for following five consecutive classifiers is smaller than or equal to that of classifier *i*, the process is stopped and classifier i is selected as the best classifier. Otherwise, Steps 3 and 4 are repeated. 5. Prediction - Base on the best classifier, the predicted labels and corresponding extrapolation degrees for samples in the validation set are calculated and recorded. Steps 1 to 5 is repeated 500 times, generating two matrices *L*(500×*p*) and *D*(500×*p*), which deposit the predicted labels and corresponding extrapolation degrees, respectively. Here, *p* indicates the number of samples in the validation set.
(0.29 MB TIF)

Figure S2 Prediction MCC as a function of extrapolation degree for nine datasets using *NC* classifier. The proportion of red in each pie chart represents the proportion of total validation set samples contained in that extrapolation degree category. Here ‘0’, ‘10’, ‘20’ and ‘>20’ in the X-axis mean ‘In domain’, ‘0–10% out of domain’, ‘10–20% out of domain’ and ‘more than 20% out of domain’, respectively.
(0.52 MB TIF)

Figure S3 Prediction MCC as a function of extrapolation degree for nine datasets using *SVM* classifier. The proportion of red in each pie chart represents the proportion of total validation set samples contained in that extrapolation degree category. Here ‘0’, ‘10’, ‘20’ and ‘>20’ in the X-axis mean ‘In domain’, ‘0–10% out of domain’, ‘10–20% out of domain’ and ‘more than 20%% out of domain’, respectively.
(0.50 MB TIF)
