# Supplementary figures and images for: Does Applicability Domain Exist in Microarray-Based Genomic Research?
Source: PLoS One. 2010 Jun 10;5(6):e11055. doi: 10.1371/journal.pone.0011055 (PMC2883551; doi:10.1371/journal.pone.0011055)

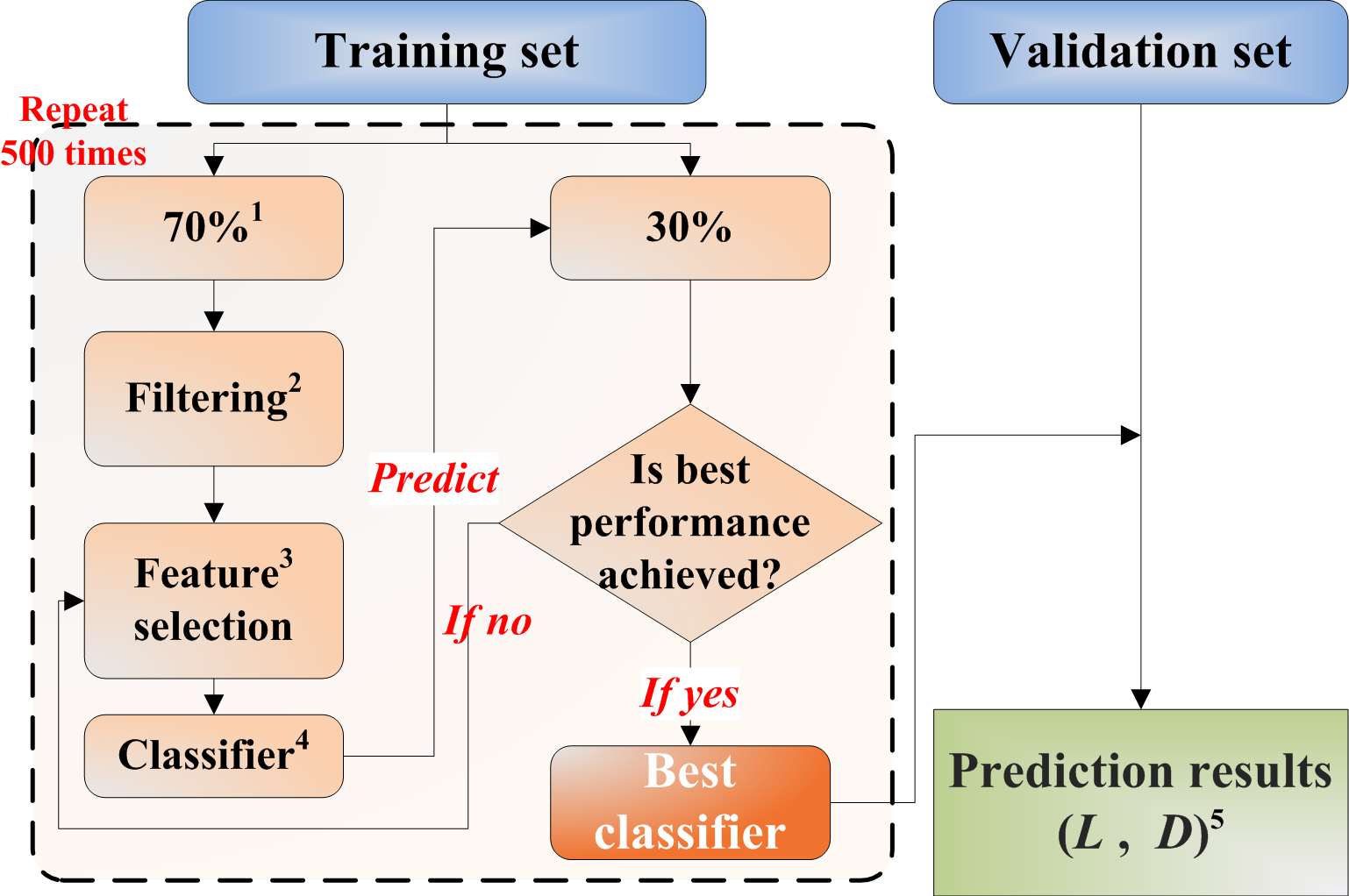

Supplement: Figure S1 — Detailed model construction procedures. The construction of the best classifier is shown as follows (see the superscripts in this figure): 1. Stratified random sample splitting - We use the 70/30 splitting, where the 70% samples are for classifier construction, and the resulting classifier is then used to predict the 30% samples to obtain the prediction performance of the classifier. To ensure statistical validity, we repeat this procedure 500 times, resulting in 500 different classifiers. 2. Filtering - This step is to generate an initial pool of probesets for further analysis. Specifically, the original pool of probesets is firstly sorted by the absolute signal-to-noise (SN) ratio, and then the 200 top ranked probesets are retained for further analysis. 3. Feature selection - We apply a sequential selection method, with the best performed probeset being sequentially added into the model to develop a classifier, which is then evaluated on the 30% samples. The process is repeated by incrementally adding one probeset at a time to generate more classifiers. 4. Classifier selection - For classifier i (i corresponds to the number of probesets selected in the classifier), if the performance MCC for following five consecutive classifiers is smaller than or equal to that of classifier i, the process is stopped and classifier i is selected as the best classifier. Otherwise, Steps 3 and 4 are repeated. 5. Prediction - Base on the best classifier, the predicted labels and corresponding extrapolation degrees for samples in the validation set are calculated and recorded. Steps 1 to 5 is repeated 500 times, generating two matrices L(500×p) and D(500×p), which deposit the predicted labels and corresponding extrapolation degrees, respectively. Here, p indicates the number of samples in the validation set. (0.29 MB TIF) [file pone.0011055.s001.tif]

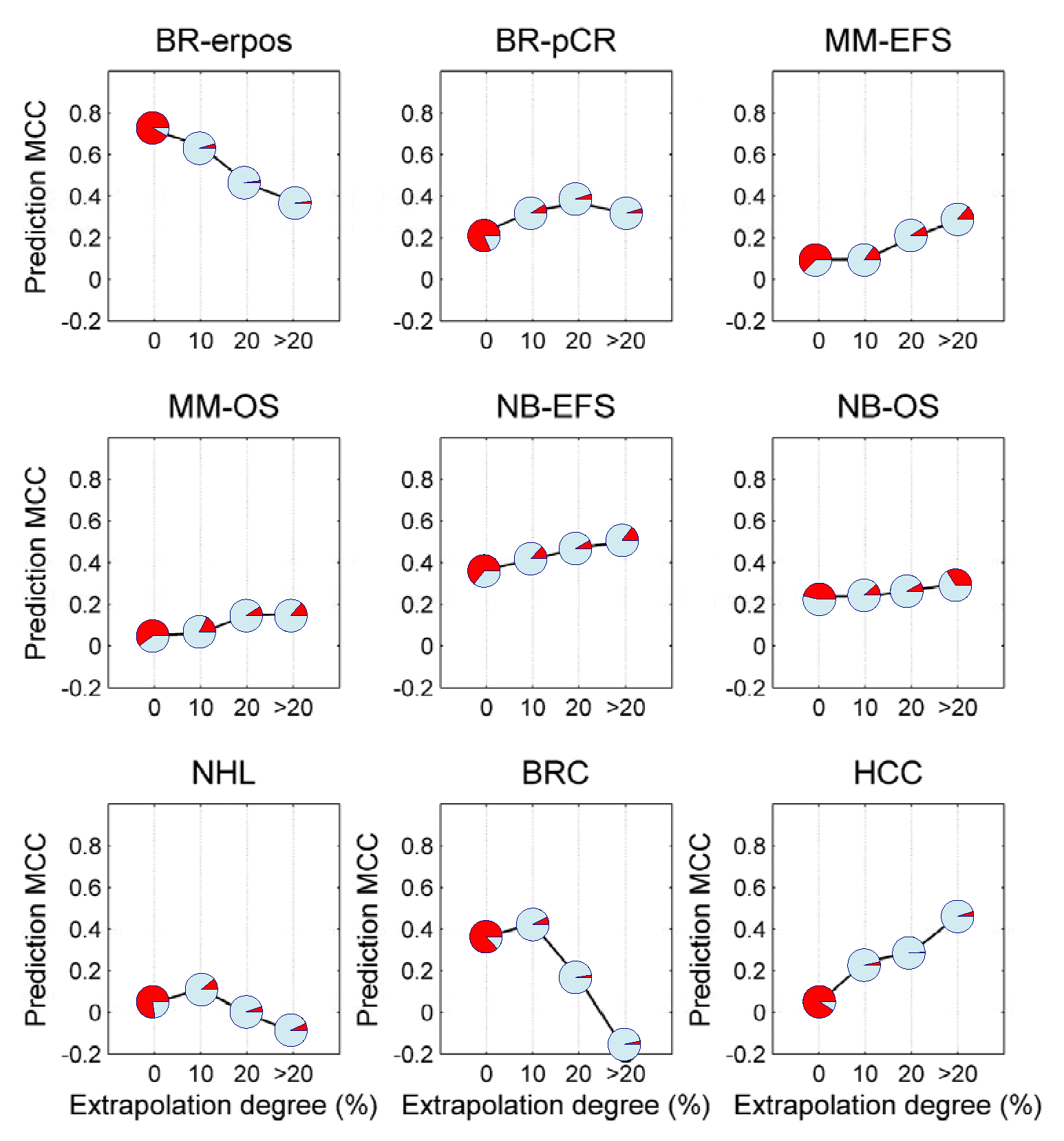

Supplement: Figure S2 — Prediction MCC as a function of extrapolation degree for nine datasets using NC classifier. The proportion of red in each pie chart represents the proportion of total validation set samples contained in that extrapolation degree category. Here ‘0’, ‘10’, ‘20’ and ‘>20’ in the X-axis mean ‘In domain’, ‘0–10% out of domain’, ‘10–20% out of domain’ and ‘more than 20% out of domain’, respectively. (0.52 MB TIF) [file pone.0011055.s002.tif]

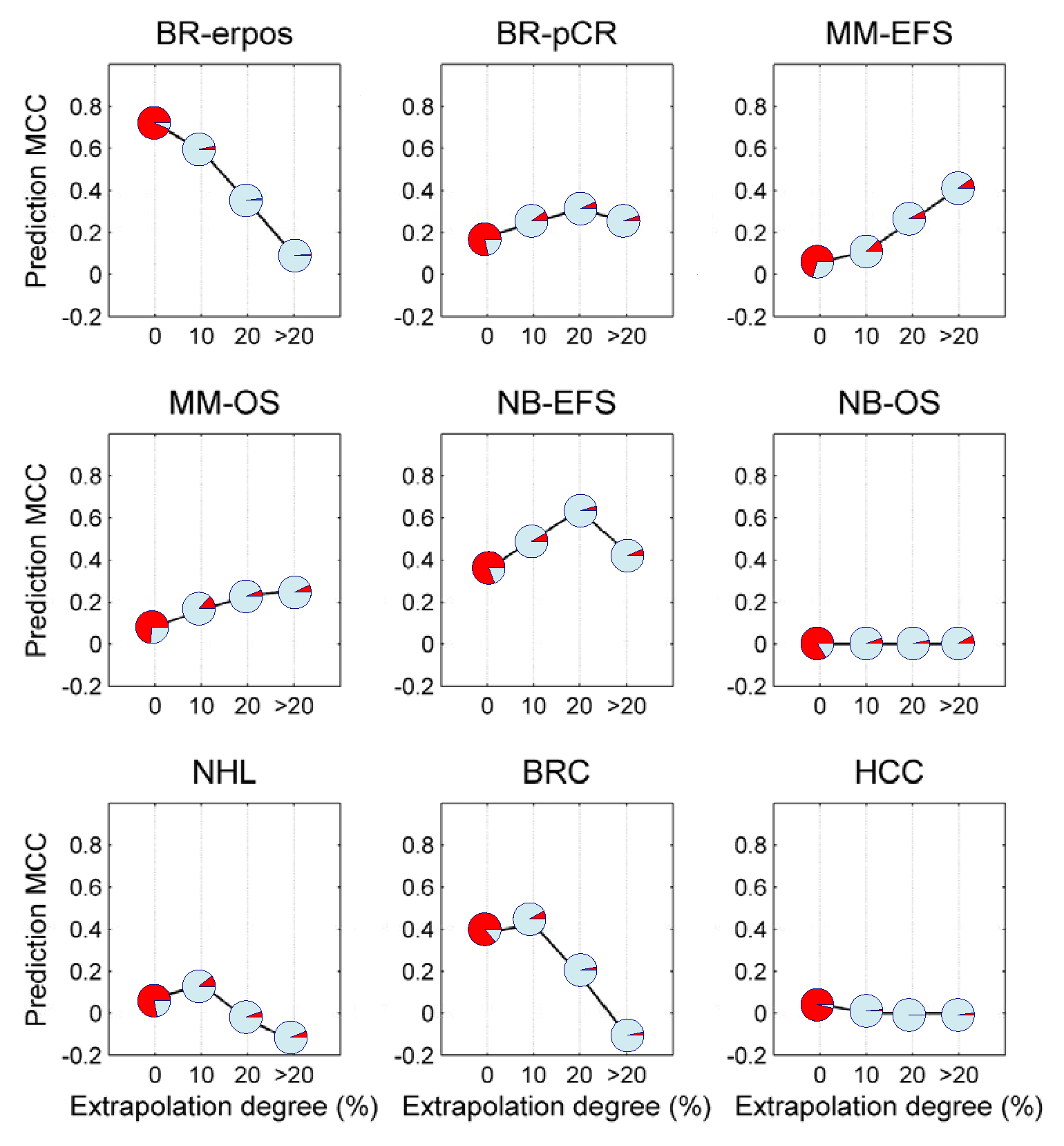

Supplement: Figure S3 — Prediction MCC as a function of extrapolation degree for nine datasets using SVM classifier. The proportion of red in each pie chart represents the proportion of total validation set samples contained in that extrapolation degree category. Here ‘0’, ‘10’, ‘20’ and ‘>20’ in the X-axis mean ‘In domain’, ‘0–10% out of domain’, ‘10–20% out of domain’ and ‘more than 20%% out of domain’, respectively. (0.50 MB TIF) [file pone.0011055.s003.tif]
